# Supplementary material for: Real-time UVMS torque distribution algorithm based on weighting matrix
Source: PLoS One. 2021 Jul 2;16(7):e0253771. doi: 10.1371/journal.pone.0253771 (PMC8253443; doi:10.1371/journal.pone.0253771)
Supplement: Attachment — Submitted filename: Response to Reviewers.pdf [file pone.0253771.s002.pdf]

To Editor

Thank you for the valuable comments on this article. We carefully revised the paper according to the comments. The main corrections are as follows:

- *This review has responded to all comments of reviewer #2. All comments from Reviewer 1 were answered in the previous review. For the readability, only the comments of Reviewer 2 are summarized.*
- *We checked and corrected grammatical errors in the paper.*
- *The sentence that confuses the understanding of the paragraph has been corrected.*
- *To increase the quality of the figure, a description was added and inserted as a vector image. Also, the color has been changed for easy identification.*
- *The contents of previous research necessary for understanding that were omitted in this paper were additionally described.*
- *Previous research contents necessary for understanding has been added.*
- *Added a description of the experimental settings.*
- *The conclusion part has been rewritten to be more specific.*
- *The reference recommended by Reviewer 2 was added to this review.*

Please refer the attached revision summary and revised draft for details. The revised contents are highlighted by colors as Yellow.

Reviewer #2

Comments #1)
The English language requires improvements. Please read the text carefully and search for typos ("a" "," "the", Capital letter, space between word, etc). For example, I fixed some part of the abstract as following that the authors could find out what I mean:
" even distributing the torque "
"For the redundant resolution of the underwater vehicle "
"Analysis of the experimental results revealed that the manipulator torque load was greatly reduced due to the AUV load distribution. "

Please spell out acronyms the FIRST time you use them in the abstract AND in the body of the manuscript, which means from the Introduction onwards. Please spell out acronyms the FIRST time you use them in the body of the manuscript again, even if already spelled out in the abstract or title.

Answer)
Thank you so much for your feedback. We have prepared different English grammar checkers and made several modifications to the typo. And we checked and corrected the acronyms you pointed out. In addition to what you pointed out, English grammar errors were checked and corrected throughout the paper.

After correction

manipulator, the error is corrected through modeling based on the Denavit–Hartenberg (DH) model [17], S Model [18], complete and parametrically continuous (CPC) model [19], Zero reference [20], and product of exponentials (POE) [21]. Non-kinematic errors are too diverse and difficult to solve [22] [23]. Non-kinematic actuator faults [12]. Cooperation of the AUV and the manipulator is effective in reducing the load on the theoretical target value. The manipulator control process consists of manipulator trajectory generation, proportional differential (PD) control, dynamics analysis, driving angle, and total angle conversion using the AUV, which can improve the overall performance. Han et al. controlled the UVMS manipulator and AUV completely to minimize the moment of restoration of the entire system [13]. According to Inoue et al., to

2.2.Desired trajectory and torque distribution

Design variable

FIGURE 7. Manipulator control flowchart

This study presents a real-time algorithm for even distributing the torque burden on the parallel manipulator with an autonomous underwater vehicle (AUV) through the cooperation of the AUV and manipulator. For the redundant resolution of the underwater vehicle manipulator system (UVMS), we used the weighting matrix of the weighted pseudo inverse for kinematic and dynamic modeling. We made dynamic and kinematic modeling using the force distribution characteristics of parallel manipulators. Using the parallel manipulator's model, the weighting matrix was changed every second to share the manipulator torque with the AUV. The Taguchi method was used to reduce the calculation time for real-time calculation and to perform valve rotation operations with as little torque as possible even in an underwater environment where it is difficult to determine any cause of errors. To demonstrate the effectiveness of this algorithm, we experimented with valve rotation in water using the UVMS. Analysis of the experimental results revealed that the manipulator torque load was greatly reduced due to the AUV load distribution.

Keywords: Cleaning devices, High-pressure, Reaction force, Optimal parameter design, Orthogonal array, Sensitivity analysis, Window contamination evaluation.

Reviewer #2

Comments #2)
"We studied this cooperation of the underwater manipulator and the AUV" you should motivate why you say that, because it does not clearly follow out of the literature discussion that you have given.

Answer) This paragraph is intended to introduce the basic studies that were conducted to make this UVMS study. The purpose of this paragraph is to provide information on previous robot development by presenting previous studies on how the AUV and manipulators of the UVMS we use were studied. It seems that the first sentence did not fully reflect this purpose, confusing the reviewer. The text has been edited to avoid confusion.

Before correction

actuator faults [12] Cooperation of the AUV and the manipulator is effective in reducing the load on the manipulator. The manipulator joint load is reduced by distributing the force using the thruster power of the AUV, which can improve the overall performance. Han et al. controlled the UVMS manipulator and AUV completely to minimize the moment of restoration of the entire system (HanJonghui & ChungWan Kyun, 2007). According to Inoue et al., to withstand the external forces, a full-body control method was applied to a mobile manipulator [11]. However, in terms of the cooperation concept, the distribution of workload between the underwater manipulator and AUV has not yet been extensively studied.

We studied this cooperation of the underwater manipulator and the AUV. In a previous study, Bae et al.

After correction

Cooperation of the AUV and the manipulator is effective in reducing the load on the manipulator. The manipulator joint load is reduced by distributing the force using the thruster power of the AUV, which can improve the overall performance. Han et al. controlled the UVMS manipulator and AUV completely to minimize the moment of restoration of the entire system [12]. According to Inoue et al., to withstand the external forces, a full-body control method was applied to a mobile manipulator [13]. However, the method of distributing the workload between joints of manipulators and the thrusters of the AUV has not been studied.

In order to present an efficient collaboration method of UVMS, we have conducted basic studies of each AUV and manipulator. In a previous study, Bae et al. studied the cooperation between an underwater

Reviewer #2

Comments #3)
Figure 10 is not clear. Please increase the quality.

Answer)
The reason Figure 10 is included is because it was decided that it would be difficult for readers to understand the structure of UVMS and the actual experimental appearance by just explaining the text. After reading your comments, we decided that it was difficult to understand what the experiment looked like compared to taking up space in the paper. So we modified it with a picture depicting the actual setup environment in the water of UVMS.

Before correction

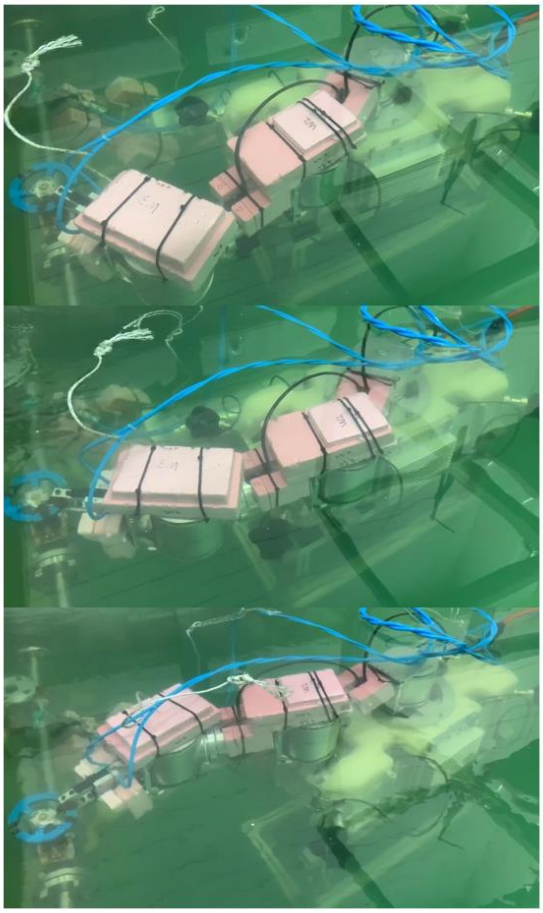

FIGURE 10. AURORA – UVMS in actual experiment

After correction

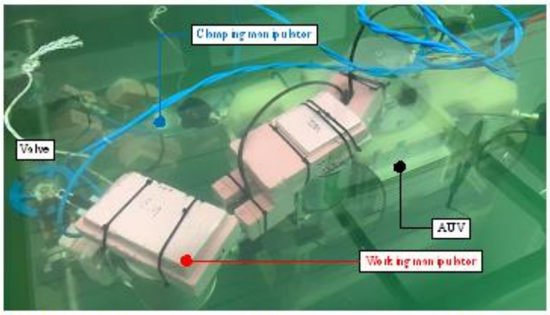

FIGURE 10. Experimental setting in the test bench

Reviewer #2

Comments #4)
Why did you select such ranges of angles?

This study deals with the difference between the conventional fixed weight matrix and the real-time weight matrix change case in the valve experiment. Therefore, the same as in the previous study, 45 degrees were selected to compare the performance difference with the previous study. If the valve is turned while the pipe is clamped, the manipulator or robot may hit the pipe. Therefore the goal was to rotate the valve 90 degrees, starting at 45 degrees where the manipulator didn't collide.

Before correction

First, we set $\dot{\boldsymbol{q}}_{V.desired}$ to the speed at which the valve will move. In this study, the valve operation was performed at 45°. The valve motion was set to the valve rotation at 1/48 π angular velocity in the counterclockwise direction, as shown in FIGURE 6.

After correction

First, we set $\dot{\boldsymbol{q}}_{V.desired}$ to the speed at which the valve will move. In this study, valve operation was performed with a motion starting at 45° and rotating through 90°. The valve motion was set to the valve rotation at 1/48 π angular velocity in the counterclockwise direction, as shown in FIGURE 6. These values are set the same value in the previous experiment in order to compare with the previous studies according to the valve work of the fixed weight matrix. [14]

Reviewer #2

Comments #5)
Quality of figures 12 ad 13 are low, and captions may be too small for the overall size of figures. Production should request higher quality figures. Color figures have been formatted so they are unclear when printed in black and white.

Answer) All the font sizes of the figures were edited and the vector image format was used so that the resolution becomes high. The color of the graph was adjusted to be easily distinguishable even when printed in black and white based on RGB.

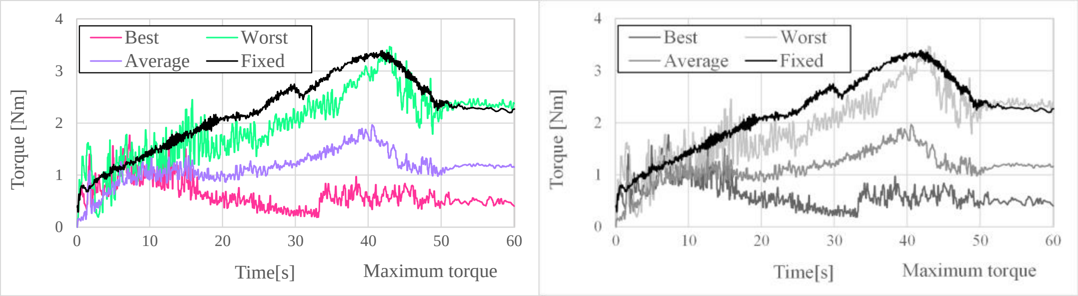

After correction

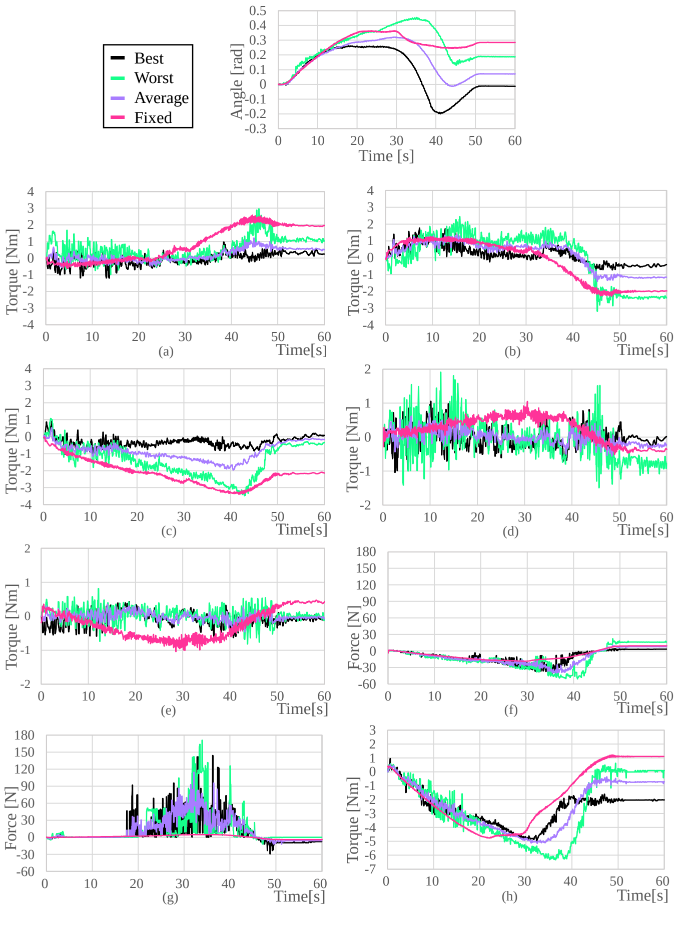

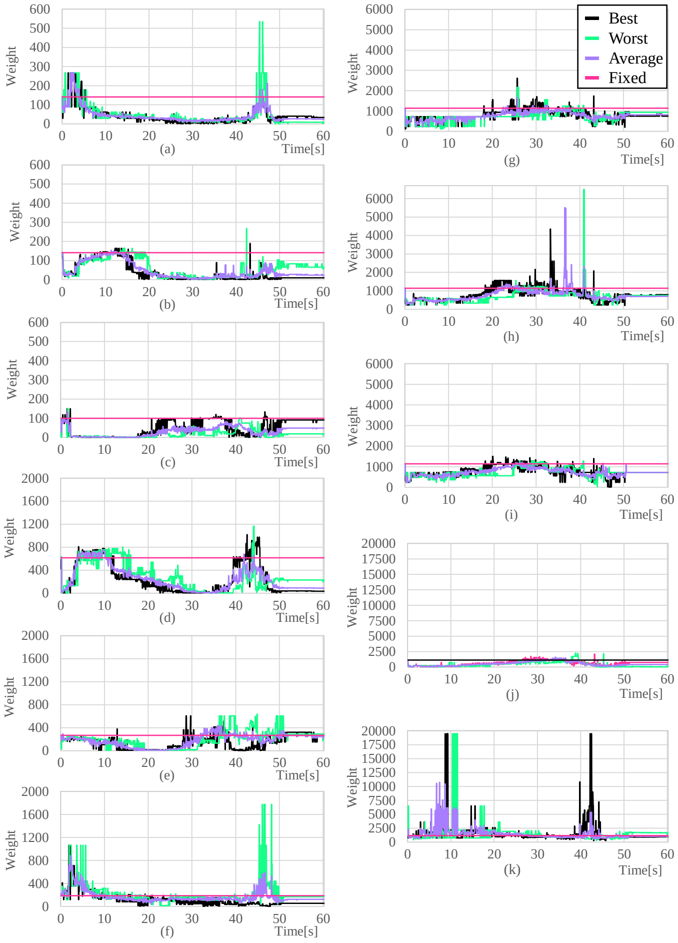

Reviewer #2

Comments #6)
"The manipulator joint torque was reduced through the distribution of the thruster's load." This is strong and contentious statement without proof? (You have not made such a case based on literature) If this is your own conclusion, it is out of place here in same paragraph with statement. And should probably be part of the next paragraph that motives the reason for your research?

Answer) As you pointed out, we found that the sentence could be confusing in a paragraph. The pointed out sentence is divided into the following paragraphs, and the explanation is described in more detail to avoid misunderstanding.

Before correction

For a more accurate comparison, we summarized the maximum torque values between the average and fixed cases. The maximum value of each joint torque during the valve operation is summarized as a bar chart in FIGURE 14, and it can be seen that all the values have decreased. The difference and the rate of change of the maximum torque and the force between the average and the fixed case are summarized in TABLE VIII and TABLE IX. The manipulator joint torque has been reduced by up to 55%. The torque of each manipulator has a very small value with a maximum of 1.4. It is because UVMS is in a neutral buoyancy state, so there is no force applied in the z-axis direction, and the force is used only to turn the valve in the xy-plane direction. The maximum thrust force of the AUV increased by 21.3 N, 109.7 N, and 0.4 Nm at F_x, F_y and, τ_ψ respectively. Owing to the limit of the thruster, the maximum range of F_x and F_y is up to 145.6 N. Therefore, the thrusters operate within the operating range. The manipulator joint torque was reduced through the distribution of the thruster's load. The torque of all the joints decreased from 38% to 55%. Compared to the fixed weight case, the sway force was significantly increased. The bent manipulator stretches as the vehicle moves backward during valve operation. The vehicle turns while pulling the valve backward. The thruster generates this backward moving force, and this force replaces the manipulator's torque. As a result, instead of decreasing the torque, the sway force in the y-axis direction increases compared to the surge force in the x-axis direction. To summarize, this algorithm sufficiently lowered the manipulator torque by distributing the manipulator's burden with the AUV's thrust.

After correction

For a more accurate comparison, we summarized the maximum torque values between the average and fixed cases. The maximum value of each joint torque during the valve operation is summarized as a bar chart in FIGURE 14, and it can be seen that all the values have decreased. The difference and the rate of change of the maximum torque and the force between the average and the fixed case are summarized in TABLE IX and TABLE X. The torque of each manipulator has a very small value with a maximum of 1.4. It is because UVMS is in a neutral buoyancy state, so there is no force applied in the z-axis direction, and the force is used only to turn the valve in the xy-plane direction. Owing to the limit of the thruster, the maximum range of F_x and F_y is up to 145.6N. Therefore, the thrusters operate within the operating range.

According to the experimental results, it can be seen that the manipulator joint torque was reduced through the distribution of the thruster load. The torque of all the joints decreased from 38% to 55% as shown in TABLE IX. The maximum thrust force of the AUV increased by 21.3N, 109.7N, and 0.4Nm at F_x, $F_y$and, τ_ψ respectively as show in TABLE X. To summarize, this algorithm sufficiently lowered the manipulator torque by distributing the manipulator's burden with the AUV's thrust. Compared to the fixed weight case, the sway force was significantly increased. The bent manipulator stretches as the vehicle moves backward during valve operation. The vehicle turns while pulling the valve backward. The thruster generates this backward moving force, and this force replaces the manipulator's torque. As a result, instead of decreasing the torque, the sway force in the y-axis direction increases compared to the surge force in the x-axis direction.

Reviewer #2

Comments #7)
"The subscript desired represents the theoretical target value" What are the other feasible alternatives? What are the advantages of adopting this particular metric over others in this case? How will this affect the results? More details should be furnished.

Answer) In a previous study, we compared the results by suggesting three alternatives to turn the valve. We used the method we judged to be the best in previous studies. It would be good to refer to the reference for the result of that method. It seemed that the explanation was omitted in the thesis, so the explanation was corrected.

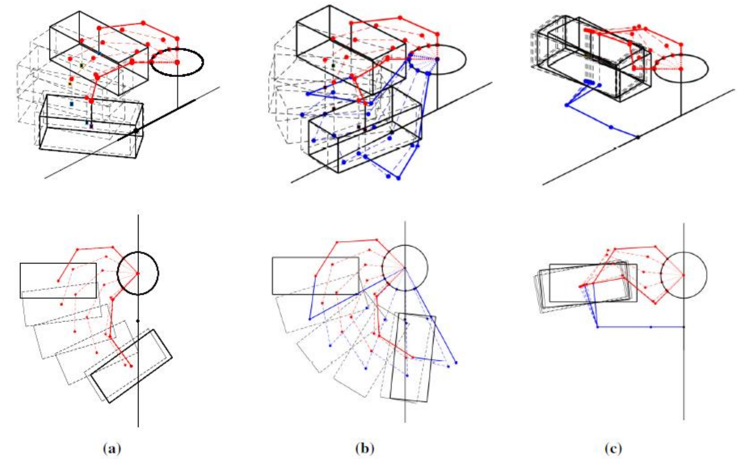

Fig. 9 Desired trajectories of the three manipulation methods derived using the kinematics equation. Initial and final configurations are depicted using thick lines. Red and blue lines, respectively, denote linkages of the working and clamping manipulators. The vehicle is denoted by the black-colored box shape. **a** Vehicle trajectory in method M1; **b** vehicle trajectory in method M2; and **c** vehicle trajectory in method M3. (Color figure online)

And the angle of the valve and the joints of the manipulator are derived through the optimization process in previous studies. Since the details have already been studied in previous papers, this paper will only mention them.

4.2.2. Desired trajectory generation of independent joints

From the desired handle valve angle trajectory, desired trajectories of independent joints were derived. To find optimal trajectory for turning handle valve, the trajectory that minimizes velocity norm of actuated joints was considered as optimal. The objective function is as follows:

$$\min(\|\dot{q}_r\|) \tag{4.11}$$

The square of velocity norm of actuated joint can be transformed as following form.

$$\|\dot{q}_r\|^2 = \dot{q}_u^T \Gamma^T \Gamma \dot{q}_u \tag{4.12}$$

To minimize this term, weighted pseudoinverse was applied to equation (4.2) as follows:

$$\dot{q}_{u,desired} = \mathbf{J}_f^{\ddagger} \dot{q}_{V,desired} \tag{4.13}$$

$$\mathbf{J}_f^{\ddagger} = (\Gamma^T\Gamma)^{-1}(\mathbf{J}_f(\Gamma^T\Gamma)^{-1})^{\dagger} \tag{4.14}$$

With the weighted pseudoinverse with weighting matrix $\Gamma^T\Gamma$, which is constraint Jacobian matrix of the system, the objective function for the trajectory (4.12) can be achieved [36]. The desired independent joint trajectory that obtained by equation (4.13) can minimize the velocity norm of all actuated joints.

Reviewer #2

Comments #7)
"The subscript desired represents the theoretical target value" What are the other feasible alternatives? What are the advantages of adopting this particular metric over others in this case? How will this affect the results? More details should be furnished.

Answer) (continued)

Before correction

2.2.Desired trajectory and Torque distribution

The control process of the manipulator is summarized in FIGURE 7. The subscript *desired* represents the theoretical target value. The manipulator control process consists of manipulator trajectory generation, PD control, dynamics analysis, driving angle, and total angle conversion using the constrained Jacobian.

First, we set $\dot{\boldsymbol{q}}_{V.desired}$ to the speed at which the valve will move. In this study, the valve operation was performed at 45°. The valve motion was set to the valve rotation at 1/48 π angular velocity in the counterclockwise direction, as shown in FIGURE 6.

After correction

2.2.Desired trajectory and torque distribution

The control process of the manipulator is summarized in FIGURE 5. The subscript *desired* represents the theoretical target value. These desired values are based on previous studies comparing the method of turning the valve based on the joint torque. [41] In the previous study, three alternatives were proposed, and among them, the method of holding the valve and pipe was pointed out as the best method. In addition, angles of the valve and joint were calculated as optimal values with a trajectory that minimizes the velocity norm in the previous study. The detailed discussion on this is described in Bae's dissertation. In this study, experiments are conducted based on the best method identified. The manipulator control process consists of manipulator trajectory generation, proportional differential (PD) control, dynamics analysis, driving angle, and total angle

Reviewer #2

Comments #8)
All the basic equations, therefore, an appropriate reference is necessary. the values of some of the parameter are not stated.

Answer) Since this study is a follow-up study, the descriptions in the previous research papers were omitted to avoid redundant descriptions. However, it is difficult to read the papers as previous research papers have not been published. Basic descriptions and references have been added so as not to interfere with understanding of this paper.

Before correction

torque that can occur during valve operation and it is possible to reduce the possibility of errors due to slip of the gripper. Even in actual operation, no-slip occurred in the gripper. The manipulator that grabs and turns the valve is called the working manipulator. Another manipulator that clamps a fixed pipe is called the clamping manipulator. This manipulator is a parallel manipulator because the manipulator forms a closed curve. $\boldsymbol{q}_w$ (q_{w1}, q_{w2}, q_{w3}) is the angle of each working manipulator. $\boldsymbol{q}_c(q_{c1}, q_{c2}, q_{c3})$is the angle of the clamping manipulator. The lengths of the links connecting each joint are expressed as l_{w1},l_{w2},l_{w3},l_{c1},l_{c2}, and l_{c3}. The position and orientation of the body can be calculated by assuming a virtual joint with 6 degrees of freedom based on the ground, which is expressed as $\boldsymbol{\eta}$. The angle of the valve rotated is denoted by q_V. The features of these joints are summarized in TABLE I.

$\mathbf{M}_v, \mathbf{M}_m$ are the inertia matrices with added mass terms of the AUV and the manipulator. $\mathbf{C}_v(\boldsymbol{v}), \mathbf{C}_m(\boldsymbol{q})$ are the centrifugal and Coriolis force matrices of the AUV and manipulator. $\boldsymbol{g}_v, \boldsymbol{g}_m$ are the gravity and buoyancy vector of the AUV and manipulator.

After correction

torque that can occur during valve operation and it is possible to reduce the possibility of errors due to slip of the gripper. Even in actual operation, no-slip occurred in the gripper. The manipulator that grabs and turns the valve is called the working manipulator. Another manipulator that clamps a fixed pipe is called the clamping manipulator. This manipulator is a parallel manipulator because the manipulator forms a closed curve. Each joint is W1, W2, W3, C1, C2 and C3 and shown in FIGURE 1. $\boldsymbol{q}_w$ (q_{w1}, q_{w2}, q_{w3}) is the angle of each working manipulator. $\boldsymbol{q}_c(q_{c1}, q_{c2}, q_{c3})$is the angle of the clamping manipulator. The lengths of the links connecting each joint are expressed as l_{w1},l_{w2},l_{w3},l_{c1},l_{c2}, and l_{c3}. l_{wn} means length between joints W_n and W_{n+1}. l_{cn} means length between joints C_n and C_{n+1}. The position and orientation of the body can be calculated by assuming a virtual joint with 6 degrees-of-freedom based on the ground, which is expressed as $\boldsymbol{\eta} = [x\ y\ z\ \phi\ \theta\ \psi]^T$. The angle of the valve rotated is denoted by q_V. The features of these joints are summarized in TABLE I.

v, m are used as a subscript means AUV and manipulator. $\mathbf{M}_v, \mathbf{M}_m$ are the inertia matrices with added mass terms. $\mathbf{C}_v(v), \mathbf{C}_m(\boldsymbol{q})$ are the centrifugal and Coriolis force. $\mathbf{D}_v(v), \mathbf{D}_m(\boldsymbol{q})$ are the hydrodynamic damping matrices. $\boldsymbol{g}_v, \boldsymbol{g}_m$ are the gravity and buoyancy vector.

Reviewer #2

Comments #9)
The measures method in experimental study are in my opinion not clear. Please use a clear definition.

Answer) There was no mention of the factors and sensors to be measured in the description of the experimental settings. What to measure in the experiment, the sensor specifically used, and the specific details of the test bench were additionally described.

Before correction

4.1. Experiment setup

To show that the algorithm is effective in real situations, experiments were conducted using robots and test benches used in previous studies. The test bench pipe was attached using a suction device on the wall of the glass tank. The overall view of the test bench is shown in FIGURE 9.The valve is located at the center of the pipe. If the valve is in the water for a long period, it can rust. Rust increases the friction in the valve rotation operation, which is an error factor in the valve operation. The actual UVMS experiment is shown in FIGURE 10.

After correction

4. Experiment and results analysis

4.1. Experiment setup

To show that the algorithm is effective in real situations, experiments were conducted using robots and test benches used in previous studies. The test bench pipe was attached using a suction device on the wall of the glass tank. The overall view of the test bench is shown in FIGURE 8. The valve is located at the center of the pipe. If the valve is in the water for a long period, it can rust. Rust increases the friction in the valve rotation operation, which is an error factor in the valve operation. The actual UVMS experiment is shown in FIGURE 9 and the position of the valve, clamping point, and diameter of the valve are depicted in FIGURE 6.

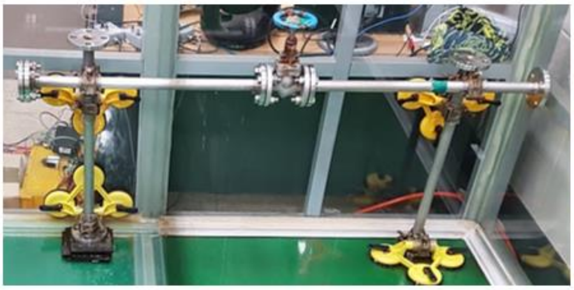

FIGURE 8. Test bench and valve structures

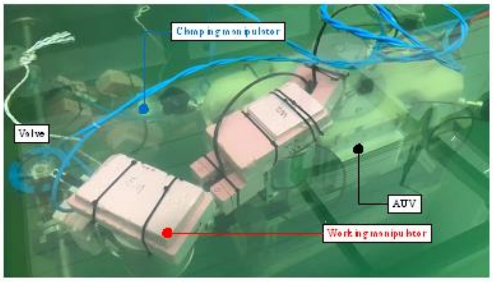

FIGURE 9. Experimental setting in the test bench

Measurements during valve operation are the angular error and torque value of each joint. It is important to ensure that the valve operation is in the desired trajectory. Therefore, the error is calculated by subtracting the desired joint angle from the current joint angle. An encoder is combined with the joint motor of the manipulator, and the encoder measures the current joint angle. The encoder is built into the Maxon EC 60 flat. The joint torque sensor measures the workload on the manipulator's joints during valve operation. The torque sensor is connected to the motor inside the joint and measures the degree of twist. The torque sensor is a special order product of SETEC, and the maximum measurable torque is about 20Nm. The internal structure of the joint is depicted in FIGURE 10. The length of each link of the UVMS used in the experiment is depicted in TABLE VI.

TABLE VI Length of UVMS links

l_{w1}	l_{w2}	l_{w3}	l_{c1}	l_{c2}	l_{c3}
350 mm	250 mm	288 mm	382 mm	463 mm	423 mm

Reviewer #2

Comments #10)
The conclusion section is too general, please add the main conclusion of the simulation studies..

Answer) As a result of referring to your comments and attached references, we decide that the existing conclusion was too general. Therefore, the conclusion was rewritten by adding the analysis through the range of the specific joint torque, thruster thrust, and weight matrix shown in the actual experiment.

The reason that the actual experimental results, not simulations, are summarized is to see the results of the algorithm in various underwater environments that are difficult to predict. For example, during UVMS testing, if the valve was immersed in water for more than 6 months, the valve had a lot of rust and the friction was strong. As another example, the thrust force of an AUV is affected by obstacles such as surrounding tank walls or pipes, and changes in the viscosity of water caused by aquatic microorganisms change. It is almost impossible to simulate all the different environments, and vice versa, generality studies are low. The purpose of this study is to present a robust algorithm that can perform well even under these unpredictable factors. Therefore, experiments were conducted using a tank in this real environment and the results were compared.

After correction

5. Conclusion

An algorithm for lowering the sum of squared torques of the manipulator joints using the Taguchi method to divide the manipulator torque with the thruster force of the AUV was presented and experimentally verified. The design variables and S/N ratio of the Taguchi method were used in real-time every second to minimize the torque sum of squares and reduce the calculation time. The optimal weighting matrix that can minimize the sum of squares of torque is repeatedly searched every second, and the control is performed to minimize the sum of squares of torque of the manipulator's joint.

To compare the fixed weighting matrix and the optimal weighting matrix selection algorithm, an experiment was conducted to measure the joint torque and thrust force of the AUV while rotating the underwater valve by 90°. Through the experiment, the error of the angle that occurs during valve operation is reduced by an average of 75%. The thrust force of AUV increased by 114.9% in the x-axis direction and 2268.3% in the y-axis direction, and the torque of each joint decreased by 53%, 38%, 42% for the working manipulator and 35%, 55% for the clamping manipulator for each joint. In addition, the weight value of the Z-axis of the AUV, which has the smallest range of the optimal weight matrix found through the search, is from 0 to 200. The weight value of manipulator joint C2, which has the largest range of the optimal weight matrix found through the search, is from 0 to 20000. It means that there is a limit with the fixed weighting matrix to cooperative control between AUV and manipulator.

A future research plan is to control using a criterion other than the sum of squares of each torque. It is expected that better performance can be achieved by modifying the criteria and control method of the optimal weighting matrix such as fuzzy logic according to the absolute torque value.

Reviewer #2

Comments #11)
the manuscript could be substantially improved by relying and citing more on recent literatures about Experimental case studies and numerical modelling such as the followings:
https://doi.org/10.1016/j.oceaneng.2020.108455, ,
https://doi.org/10.1115/1.4049040,
https://doi.org/10.1016/j.engfailanal.2020.104548,
https://doi.org/10.1063/1.5113592,
https://doi.org/10.1016/j.flowmeasinst.2020.101717

Answer) The content on non-kinematic errors in the introduction was supplemented by citing the content on modeling, and experimental papers were added to the description of the experiment.

Before correction

There are many geometric and non-geometric compensation methods for manipulator errors; however, it is difficult to apply these techniques to underwater manipulators. To correct the geometric error in the manipulator, the error is corrected through modeling based on the Denavit–Hartenberg (DH) model [19], S Model [20], complete and parametrically continuous (CPC) model [21], Zero reference [22], and product of exponentials (POE) [23]. Non-kinematic errors are too diverse and difficult to solve [24] [25]. Non-kinematic errors have been studied in specific fields such as the deformation of gears [26], links, and joints [27]. In addition, laser trackers [28], 3D measuring devices [29], and cameras [30] that measure the errors in these methods are difficult to use underwater because of their large external measuring devices. To summarize, it is difficult to correct and measure the cause of torque difference in an underwater manipulator. Therefore, the joint torque should be lowered without modeling.

In this study, a real-time algorithm that divides the manipulator's torque load with an AUV through the cooperation of the AUV and the manipulator was proposed and tested. To reduce the computation time, we used the DOE and the S/N ratios. By changing the weighting matrix using the manipulator joint angle and model every second, a weighting matrix element with the highest S/N ratio was determined. Even if the underwater situation changes rapidly and is unknown, the weighting matrix is calculated based on the Taguchi method at every moment. This weighting matrix is selected in the best-case scenario, and then the torque calculation and manipulator control are performed. An experiment was conducted to prove the effectiveness of this algorithm by comparing it with the performance of the valve operation used in the previous Bae's study. This experiment was conducted in an underwater environment of a large tank filled with water by attaching an actual commercial valve and pipe to the tank. In the experiment, the valve was rotated 90°, similar to Bae's study, and the torque was compared with the case in the previous study where the weighting matrix was fixed. Even if the specific error factor is unknown, DOE and the level average analysis of DOE are executed in real-time, so that the torque can be distributed efficiently.

After correction

There are many geometric and non-geometric compensation methods for manipulator errors; however, it is difficult to apply these techniques to underwater manipulators. To correct the geometric error in the manipulator, the error is corrected through modeling based on the Denavit–Hartenberg (DH) model [19], S Model [20], complete and parametrically continuous (CPC) model [21], Zero reference [22], and product of exponentials (POE) [23]. Non-kinematic errors are too diverse and difficult to solve [24] [25]. Non-kinematic errors have been studied in specific fields such as the deformation of gears [26], links, and joints [27]. In addition, laser trackers [28], 3D measuring devices [29], and cameras [30] that measure the errors in these methods are difficult to use underwater because of their large external measuring devices. Similarly, in fluid mechanics, problems that are difficult to solve are often modeled through analysis because there are many factors to consider [31] [32]. However, in large-scale multi-system UVMS, it is difficult to apply due to the limitations of the analysis device. To summarize, it is difficult to correct and measure the cause of torque difference in an underwater manipulator. Therefore, the joint torque should be lowered without modeling.

In this study, a real-time algorithm that divides the manipulator's torque load with an AUV through the cooperation of the AUV and the manipulator was proposed and tested. To reduce the computation time, we used the DOE and the S/N ratios. By changing the weighting matrix using the manipulator joint angle and model every second, a weighting matrix element with the highest S/N ratio was determined. Even if the underwater situation changes rapidly and is unknown, the weighting matrix is calculated based on the Taguchi method at every moment. This weighting matrix is selected in the best-case scenario, and then the torque calculation and manipulator control are performed. An experiment was conducted to prove the effectiveness of this algorithm by comparing it with the performance of the valve operation used in the previous Bae's study. This experiment was conducted in an underwater environment of a large tank filled with water by attaching an actual commercial valve and pipe to the tank. In the experiment, the valve was rotated 90°, similar to Bae's study, and the torque was compared with the case in the previous study where the weighting matrix was fixed. Even if the specific error factor is unknown, DOE and the level average analysis of DOE are executed in real-time, so that the torque can be distributed efficiently. To organize the results of the experiment, we refer to papers that analyze the results by changing variables such as speed and angle using commercially available products [37] [38] [39].

[31] H. Jordaan, P. Stephan Heyns and S. Hoseinzadeh, "Numerical Development of a Coupled One-Dimensional/Three-Dimensional Computational Fluid Dynamics Method for Thermal Analysis With Flow Maldistribution," *Journal of Thermal Science and Engineering Applications,* vol. 13, no. 4, 2021.

[32] S. Hoseinzadeh and P. S. Heyns, "Thermo-structural fatigue and lifetime analysis of a heat exchanger as a feedwater heater in power plant," *Engineering Failure Analysis,* vol. 113, 220.

[33] A. Van Schepdael, A. Carlier and L. Geris, "Sensitivity Analysis by Design of Experiments," in *Studies in Mechanobiology, Tissue Engineering and Biomaterials*, vol. 17, Springer, 2016, pp. 327-366.

[34] R. K. Roy, A primer on the Taguchi method, Society of Manufacturing Engineers, 2010.

[35] H. Lim, S. Hwang, K. Shin and C. Han, "The application of the Grey-based Taguchi method to optimize the global performances of the robot manipulator," *IEEE/RSJ 2010 International Conference on Intelligent Robots and Systems, IROS 2010 - Conference Proceedings,* pp. 3868-3874, 2010.

[36] P. Nikdel, M. Hosseinpour, M. A. Badamchizadeh and M. A. Akbari, "Improved Takagi-Sugeno fuzzy model-based control of flexible joint robot via Hybrid-Taguchi genetic algorithm," *Engineering Applications of Artificial Intelligence,* vol. 33, pp. 12-20, 2014.

[37] S. Hoseinzadeh, A. Sohani and S. Heyns, "Comprehensive analysis of the effect of air injection on the wake development of an airfoil," *Ocean Engineering,* vol. 220, 2021.

[38] A. Bahrami, S. Hoseinzadeh, P. S. Heyns and S. M. Mirhosseini, "Experimental investigation of co-flow jet's airfoil flow control by hot wire anemometer," *Review of Scientific Instruments,* vol. 90, no. 12, 2019.

[39] S. Hoseinzadeh, A. Bahrami, S. M. Mirhosseini, A. Sohani and S. Heyns, "A detailed experimental airfoil performance investigation using an equipped wind tunnel," *Flow Measurement and Instrumentation,* vol. 72, 220.
